# Exosome‐mediated delivery of miR‐204‐5p inhibits tumor growth and chemoresistance

**DOI:** 10.1002/cam4.3248

**Published:** 2020-07-02

**Authors:** Surui Yao, Yuan Yin, Guoying Jin, Dan Li, Min Li, Yaling Hu, Yuyang Feng, Yuhang Liu, Zehua Bian, Xue Wang, Yong Mao, Jia Zhang, Zhimeng Wu, Zhaohui Huang

**Affiliations:** ^1^ Wuxi Cancer Institute Affiliated Hospital of Jiangnan University Wuxi Jiangsu China; ^2^ Laboratory of Cancer Epigenetics Wuxi School of Medicine Jiangnan University Wuxi Jiangsu China; ^3^ Department of Oncology Affiliated Hospital of Jiangnan University Wuxi Jiangsu China; ^4^ Key Laboratory of Carbohydrate Chemistry & Biotechnology Ministry of Education School of Biotechnology Jiangnan University Wuxi China

**Keywords:** cancer therapy, chemoresistance, colorectal cancer, drug delivery, exosome, miR‐204‐5p

## Abstract

**Background:**

Nano‐sized extracellular vesicles secreted by cells play key roles in intercellular crosstalk, and appear to be an excellent biocompatible material as therapeutic cargoes in vivo. Previously, we have demonstrated that miR‐204‐5p is a key tumor suppressor that could inhibit tumor growth, metastasis and chemoresistance.

**Methods:**

A HEK293T cell line stably expressing miR‐204‐5p (293T‐miR‐204) was constructed by lentivirus transduction. Fluorescence real‐time quantitative PCR (qPCR) was applied to measure the expression of miR‐204‐5p. CCK‐8 and colony formation assays were used to evaluate the in vitro anticancer effects, and the flow cytometry was used to detect apoptosis. The in vivo therapeutic effects of exosomal miR‐204‐5p were evaluated using a xenograft mouse model. Western blots were used to detect the protein levels of CD63, Flotillin‐2, RAB22A and Bcl2. The protein levels of RAB22A and Bcl2 in tumor tissues were measured by immunohistochemistry staining.

**Results:**

MiR‐204‐5p was clearly upregulated in CRC cells after coculturing with 293T‐miR‐204 cell‐derived conditioned medium (CM) or exosomes. CCK‐8 and colony formation assays showed that the cell proliferation ability of CRC cells was clearly inhibited by 293T‐miR‐204 cell‐derived CM or exosomes. The inhibitory effects of exosomal miR‐204‐5p on cell proliferation were further confirmed in other types of cancers. Exosomal miR‐204‐5p could induce apoptosis and increase the sensitivity of cancer cells to the chemotherapeutic drug—5‐fluorourcil. In addition, exosomal miR‐204‐5p inhibited the tumor growth in mice. Western blot assay and IHC staining showed that the protein levels of miR‐204‐5p targets were clearly decreased in cancer cells or xenograft tissues treated with exosomal miR‐204‐5p.

**Conclusions:**

In this study, we confirmed that exosomal miR‐204‐5p could efficiently inhibit cancer cell proliferation, induce apoptosis and increase chemosensitivity by specifically suppressing the target genes of miR‐204‐5p in human cancer cells.

## Graphical abstract

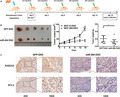

## 1 INTRODUCTION

Exosomes are nano‐sized extracellular vesicles (EVs) (30‐150 nm) that could be secreted by almost all types of cells under physiological or pathological conditions.^1^ Exosomes mediate intercellular communication between donor and recipient cells by enriching and delivering nucleic acid, protein, metabolites, or lipid from derived cells.^2^ Due to its unique characteristics, including nanoscale size, low toxicity, immune compatibility, circulation stability in vivo, exosomes appear to be an excellent biomaterial as therapeutic cargoes in vitro and in vivo.^3^, ^4^

MicroRNAs (miRNAs) are small noncoding RNAs that inhibit gene expression by binding to the 3′ untranslated regions of target mRNAs. Recent advances have showed that exosomal miRNA are important mediators of cancer‐host crosstalk, and cancer cell‐derived exosomal miRNAs could regulate the functions and phenotypes of distant cells, especially by promoting crosstalk among various cells in tumor microenvironment. Exosomes are not only stable in structure that can protect their contents from degradation, but also have the advantages of noncytotoxicity, low immunogenicity and high biocompatibility. Therefore, exosome is considered to be a new ideal drug delivery carrier. Recently, exosomes were used to transport therapeutic miRNAs to silence the corresponding target genes in recipient cells. Let‐7a, a tumor suppressor miRNA, were delivered by exosomes to inhibit EGFR in breast cancer xenografts.^5^ MSC‐derived exosomes loading with miRNA‐143 were transferred to osteosarcoma cells and could inhibit their migration.^6^ Exosomal miR‐146b from marrow stromal cells were used to reduce glioma xenograft growth in a rat model of primary brain tumor.^7^

We previously revealed that miR‐204‐5p is frequently silenced in colorectal cancer (CRC) and could inhibit tumor growth, metastasis, and chemoresistance.^8^, ^9^ Other groups also reported its tumor suppressive roles in different types of human cancer.^10^, ^11^, ^12^, ^13^ For example, miR‐204‐5p could suppress lymph node metastasis via regulating CXCL12 and CXCR4 in gastric cancer.^14^ It also inhibits tumor metastasis and immune cell reprogramming in breast cancer through the regulation of PI3K/Akt signaling.^15^ These data suggest that miR‐204‐5p is a powerful pan‐cancer suppressor and restoring its expression may be a promising strategy for human cancer therapy.^16^ In this study, we generated a HEK293T cell line that stably secretes high levels of exosomal miR‐204‐5p, and then evaluated its therapeutic value for human cancers. Our data demonstrated that exosomal miR‐204‐5p appears to be a promising novel therapeutic strategy for human cancers.

## 2 MATERIALS AND METHODS

### 2.1 Cell lines

Human cell lines, including HEK293T cells (293T), CRC cells (LoVo and HCT116), breast cancer cell (MCF‐7), glioma cell (U251), lung cancer cell (A549), gastric cancer cell (SGC‐7901), were authenticated by a short tandem repeat assay by Genewiz (China).

### 2.2 Lentivirus production and transduction

The sequence of pri‐miR‐204 was cut from pWPXL‐miR‐204^8^ and subcloned into the lentivirus expression vector pCDH‐CMV‐MCS‐EF1‐copGFP‐T2A‐Puro (pCDH‐miR‐204). PCDH‐miR‐204 plasmid or the control vector was transfected into 293T cells along with pMD2G (envelope plasmid) and ps‐PAX2 (packaging plasmid) using Lipofectamine 2000 (Invitrogen). Virus particles were collected after 48 hours, and were then used to infect 293T cells to get 293T‐GFP cells or 293T‐miR‐204 cells, respectively. These cells were then treated with puromycin (4 µg/mL) for three days and were harvested for qRT‐PCR validation.

### 2.3 Real‐time RT‐qPCR

Total RNA was extracted from cell or exosome samples using TRIzol or TRIzol LS (Invitrogen), respectively. The RNA concentrations were determined using NanoDrop 2000 (Thermo). The relative levels of miR‐204‐5p and miR‐204‐3p were measured by RT‐qPCR as we previously described.^8^

### 2.4 Preparation of the conditioned medium (CM)

293T‐GFP cells or 293T‐miR‐204 cells were plated in dishes and cultured to about 70% density. The culture medium was then replaced with the exosome‐depleted fetal bovine serum (5%) and these cells were grown for additional 48‐72 hours. The medium from each culture was then collected and subjected to centrifugation at 2000 *g* at 4℃ for 30 minutes to remove residual cell debris. These media were used for functional assays as CMs or for exosome purification.

### 2.5 Isolation and characterization of exosome

Cell‐free media derived from 293T‐GFP cells or 293T‐miR‐204 cells were centrifuged at 10 000 *g* for 60 minutes and 110 000 *g* for 70 minutes at 4°C. The supernatants were aspirated and the exosome pellets were resuspended in PBS buffer. Transmission electron microscopy (TEM) was used to observe the shape of exosomes as we previously described.^17^ Zetasizer Nano ZS (Malvern Instruments) was used to determine the size distribution of isolated exosomes. The protein concentration of the exosome samples was detected using a BCA Protein Assay kit (CWBIO). For cell treatment, equivalent to 10 μg of exosomes were added to 1 × 10^5^ recipient cells.

### 2.6 Western blot analysis

The protein samples of cells and exosomes were detected with antibodies against CD63 (1:1000; BOSTER), Flotillin‐2 (1:500; Santa Cruz), RAB22A (1:1000; Proteintech), Bcl2 (1:1000; Santa Cruz) and β‐actin (1:2000; Thermo) as we previously described.^8^, ^18^

### 2.7 Cell proliferation and colony formation assays

Cancer cells were incubated with CMs or exosomes for 3 days, and were then harvested for cell proliferation and colony formation assays. The 1000‐1500 cells were seeded in 96‐well plates and these cells were grown for additional 4 days. Cell growth activity was measured using a Cell Counting Kit‐8 (CCK‐8; Beyotime). For colony formation assays, 800‐1000 cells were seeded in each well of 6‐well plates and maintained in completed media for 10 days, and CMs or exosomes were added to the medium at day 2 and day 5. The colonies were fixed with 20% methanol and stained with 0.1% crystal violet for 30 minutes. The number of colonies was then counted.

### 2.8 Assessment of apoptosis

LoVo or HCT116 cells cocultured with CMs or exosomes were treated with 5‐FU (6 µg/mL). After 48 hours, these cells were then collected and subjected to apoptosis analyses using an Annexin V‐FITC/PI Kit (CWBIO).

### 2.9 Assessment of chemotherapy sensitivity

Cancer cells cocultured with exosomes of 293T‐GFP (GFP EXO) or 293T‐miR‐204 (miR‐204 EXO) were treated with gradually changing concentrations of 5‐fluorourcil (5‐FU), and the cell viability was then determined by CCK‐8 assays. IC50s (half‐maximal inhibitory concentrations) were calculated using Graphpad Prism.

### 2.10 In vivo anticancer effect of exosomal miR‐204 in nude mouse

To construct a subcutaneous xenograft tumor model, HCT116 cells (2 × 10^6^) were resuspended in 100 μL PBS and then subcutaneously injected into right flanks of athymic male BALB/c nude mouse. A week later, a total of 100 μg of GFP EXO or miR‐204 EXO was respectively injected into the xenograft tumors once every 3 days.^17^ The mice were sacrificed after the fifth injection. The maximum diameter (a) and minimum diameter (b) of tumor was measured, and volume was calculated by formula (V = 1/2ab^2^). Tumor tissues were weighed, fixed in 10% formalin, and then embedded in paraffin. All these procedures were performed in accordance with the Guide for the Care and Use of Laboratory Animals and Jiangnan University Institutional Ethical Guidelines for animal experiments.

### 2.11 Immunohistochemistry (IHC) staining

Tissues embedded in paraffin were cut into 5 µm sections and subjected to IHC staining. Antibodies used included anti‐RAB22A (1:200; ProteinTech), anti‐Bcl2 (1:100; Santa Cruz) as we previously described.^19^

### 2.12 Statistical analyses

Data are expressed as the mean ± SEM and were analyzed by Student's *t* test. All statistical tests were two‐sided. Any differences were considered statistically significant at *P* < .05. All analyses were performed with GraphPad Prism version 8.

## 3 RESULTS

### 3.1 Characteristics of exosomal miR‐204‐5p

**Figure 1:**
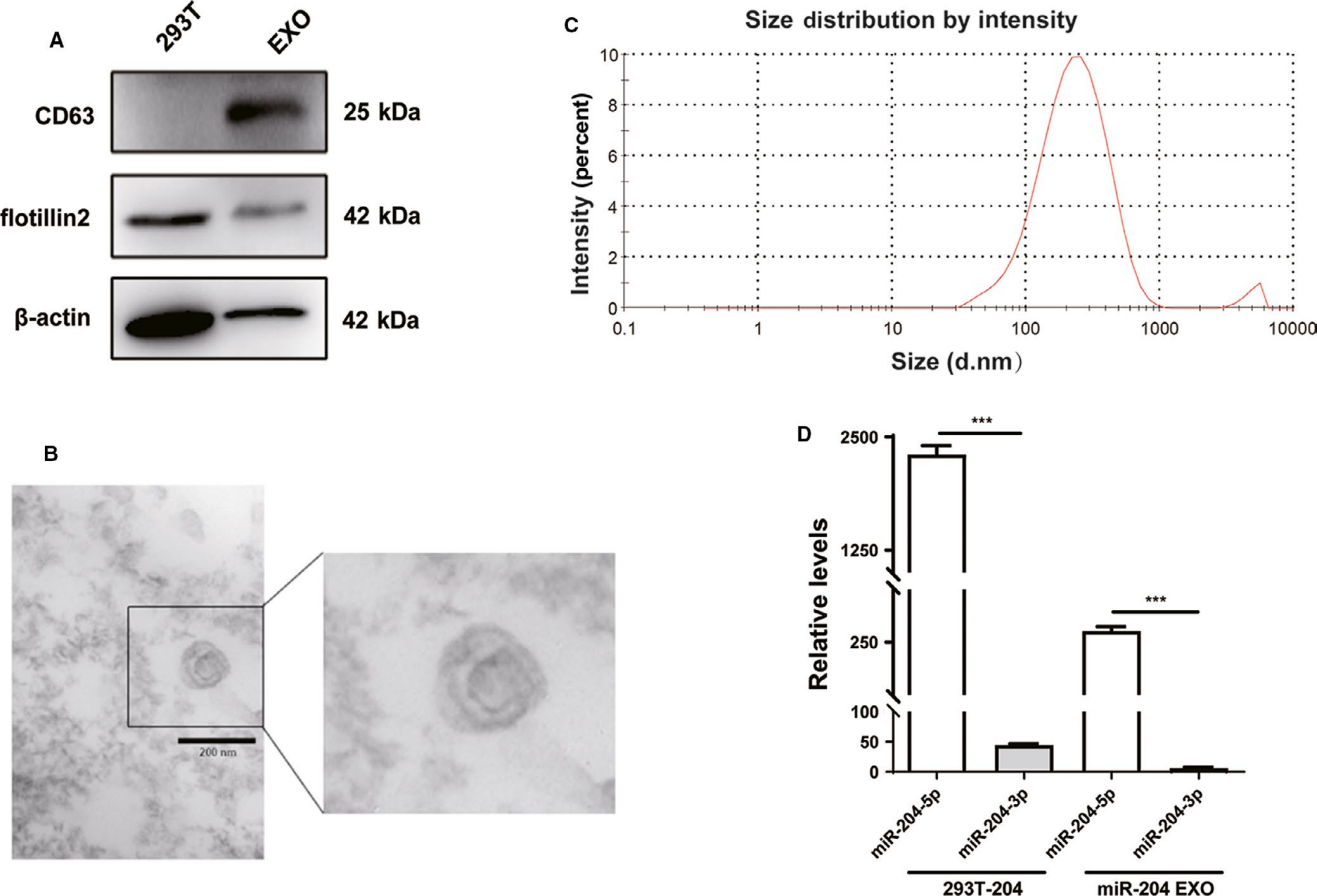
Characteristics of exosomes derived from miR‐204‐5p‐overexpressing HEK293T cells. A, The markers of exosomes (CD63 and Flotillin‐2) were detected in HEK293T cells and exosomes by Western blot. B, The transmission electron micrograph showed roundshaped vesicles with bilayered membranes ranging from 100 nm to 150 nm in diameter released by HEK293T cells. Scale bar = 200 nm. C, 293T EXOs size distribution was measured by Zetasizer. D, Real‐time qRT‐PCR revealed that the level of miR‐204‐5p was higher in 293T‐204 cells and miR‐204 EXO than miR‐204‐3p. ***P < .001. Shown are mean ± SEM from three independent experiments

We previously reported that miR‐204‐5p is frequently silenced in CRC and could inhibit tumor growth, metastasis, and chemoresistance.^8^, ^9^ To explore the therapeutic value of miR‐204‐5p for human cancers, we constructed a HEK293T cell line that stably secretes high levels of exosomal miR‐204‐5p. Exosomes were purified from culture media by the ultracentrifuge method, and the protein levels of exosome markers (CD63 and Flotillin‐2) were validated in these exosomes using Western blot (Figure 1A). In addition, when observed using TEM, exosomes appeared as small round vesicles of 100‐150 nm in diameter (Figure 1B). In addition, the Z‐Average (d.nm) of exosomes tested by Zetasizer was 194.4 nm (Figure 1C), and the hydrodynamics diameter of exosomes measured was approximately 250 nm (97.5%). The levels of miR‐204‐5p were significantly increased in 293T‐miR‐204 cells and miR‐204 EXO compared with their corresponding controls, suggesting that miR‐204‐5p was effectively packaged. Because pri‐miR‐204 could produce both miR‐204‐3p and miR‐204‐5p, the levels of miR‐204‐3p in 293T‐miR‐204 cells and their exosomes were also detected (Figure 1D). The results showed that the levels of miR‐204‐3p were much lower than miR‐204‐5p in these samples, suggesting that the effects of exosomal miR‐204‐3p on tumor cells could be neglected.

### 3.2 293T‐miR‐204‐5p CM inhibits CRC cell proliferation and induces apoptosis

**Figure 2:**
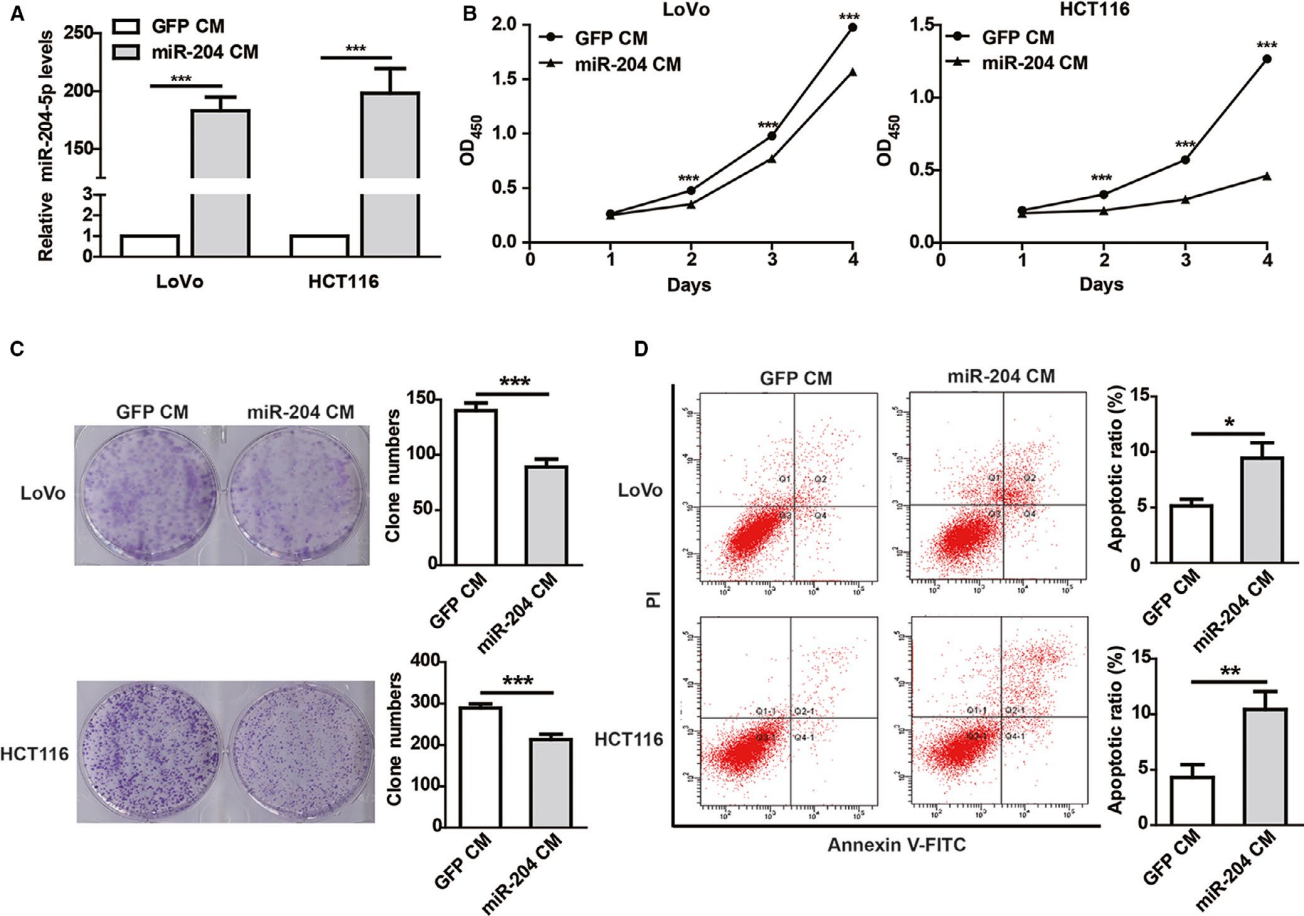
Conditioned medium (CM) of 293T‐miR‐204 cells inhibits CRC cells proliferation and induces apoptosis. A, The expression of miR‐204‐5p in CRC cells cocultured with CM of miR‐204‐5p‐overexpressing 293T cells (miR‐204 CM) or the control (GFP CM) detected by qRT‐PCR. B‐C, miR‐204 CM inhibited CRC cell proliferation (B) and colony formation ability (C) compared with the control CM. D, miR‐204 CM enhanced 5‐FU‐induced apoptosis in CRC cells. CRC cells cocultured with miR‐204 CM or GFP CM for 24 hours were treated with 6 µg/mL 5‐FU for 48 hours. Flow cytometry showed that miR‐204 CM enhanced 5‐FU‐induced apoptosis in CRC cells.*P < .05, **P < .01, ***P < .001. Shown are mean ± SEM from three independent experiments

In order to prove that the miR‐204‐5p could be transported from the donor cells (293T) into cancer cells, we cocultured colorectal cancer (CRC) cells (HCT116 and LoVo) with the CMs of 293T‐miR‐204 or 293T‐GFP cells. After coculturing with the CM, the expression of miR‐204‐5p in HCT116 and LoVo cells was observed to be clearly upregulated (~200 folds), suggesting that some components packing miR‐204‐5p in the culture supernatant of 293T‐miR‐204 cells could enter into CRC cells efficiently (Figure 2A). Functionally, the CM of 293T‐miR‐204 could significantly inhibit cell proliferation (Figure 2B) and colony formation (Figure 2C) of CRC cells compared with the control. In addition, the 5‐FU‐induced apoptosis in CRC cells cocultured with the 293T‐miR‐204 CM was significantly increased compared with the control (Figure 2D). These data suggested that 293T‐miR‐204 cells could exert tumor suppressive functions by paracrine action.

### 3.3 Exosomal miR‐204‐5p inhibits cancer cell proliferation and induces apoptosis

**Figure 3:**
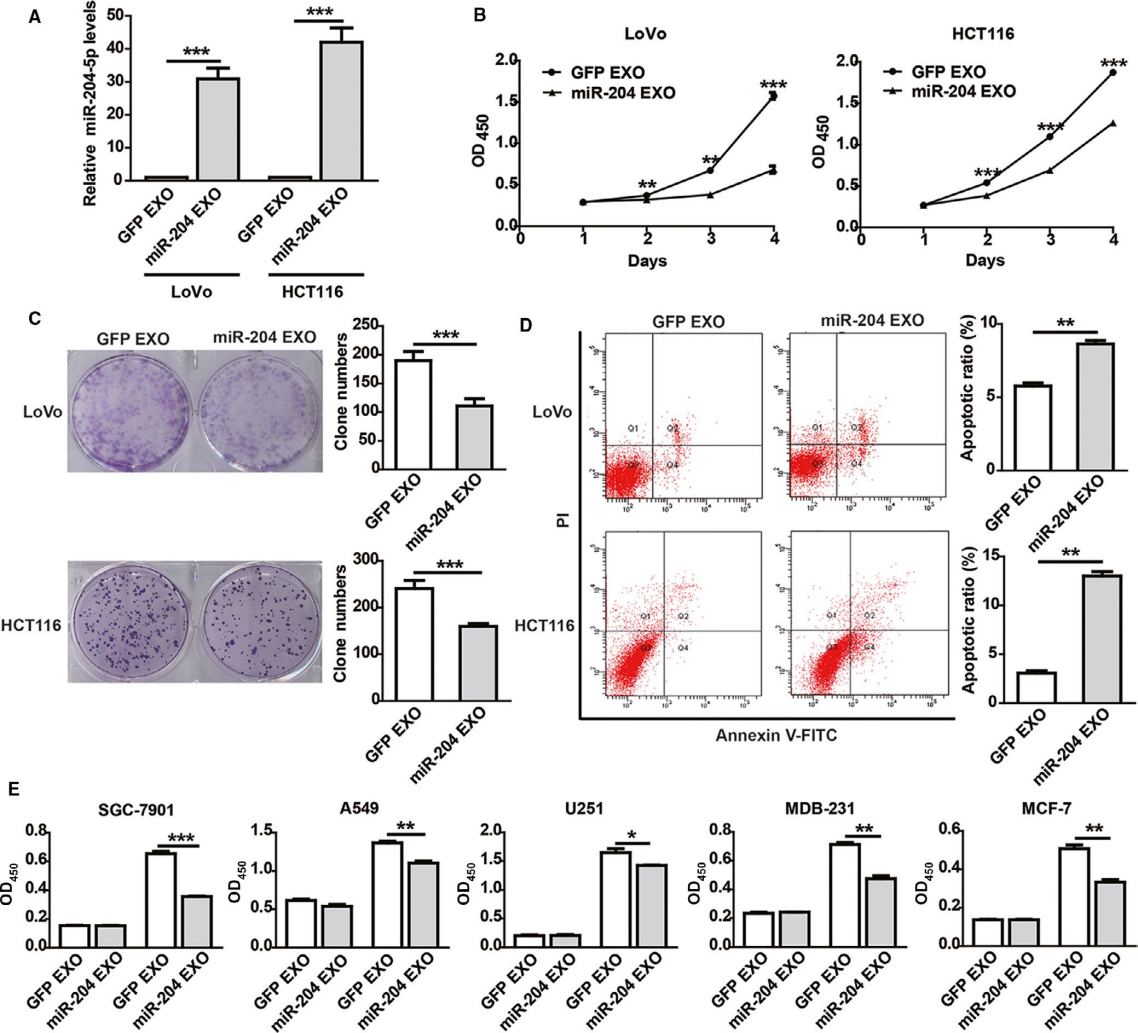
Exosomal miR‐204‐5p inhibits CRC cells proliferation and induces apoptosis. A, The expression of miR‐204‐5p in CRC cells cocultured with 293T‐derived exosomes. B‐C, Exosomal miR‐204‐5p (miR‐204 EXO) inhibited CRC cell proliferation (B) and colony formation ability(C) compared with the control exosomes (GFP EXO). D, CRC cells cocultured with miR‐204 EXO or GFP EXO for 24 hours were treated with 6 µg/mL 5‐FU for 48 hours. Flow cytometry showed that miR‐204‐5p EXO enhanced 5‐FU‐induced apoptosis in CRC cells. E, Exosomal miR‐204‐5p (miR‐204‐5p EXO) inhibited cell proliferation in breast cancer (MCF‐7 and MDB‐231), lung cancer (A549), gastric cancer (SGC‐7901), and glioma (U251) cells. The cell proliferation activity was measured by CCK‐8 at 24 hours and 72 hours after coculturing with EXO. *P < .05, **P < .01, ***P < .001. Shown are mean ± SEM from three independent experiments

To further check whether 293T‐miR‐204 cells play tumor suppressive roles through exosomal miR‐204‐5p, we cocultured CRC cells with miR‐204 EXO, and the results showed that the expression of miR‐204‐5p in these CRC cells was also clearly increased compared with the control (Figure 3A). Consequently, functional assays demonstrated that miR‐204 EXO could significantly inhibit cell proliferation and colony formation ability (Figure 3B and C) in CRC cells. The 5‐FU‐induced apoptosis of CRC cells cocultured with miR‐204 EXO was clearly increased compared with the control (Figure 3D). To further determine the tumor suppressive functions of miR‐204 EXO, we detected its effects on the cell proliferation of other cancer types, including breast cancer (MCF‐7 and MDA‐MB‐231), lung cancer (A549), gastric cancer (SGC‐7901), and glioma (U251) cells. The CCK‐8 results confirmed that miR‐204 EXO could inhibit the proliferation of these cancer cells (Figure 3E). Together, these data demonstrated that miR‐204 EXO is an efficient pan‐cancer suppressor.

### 3.4 Exosomal miR‐204‐5p inhibits the expression of RAB22A and Bcl2

**Figure 4:**
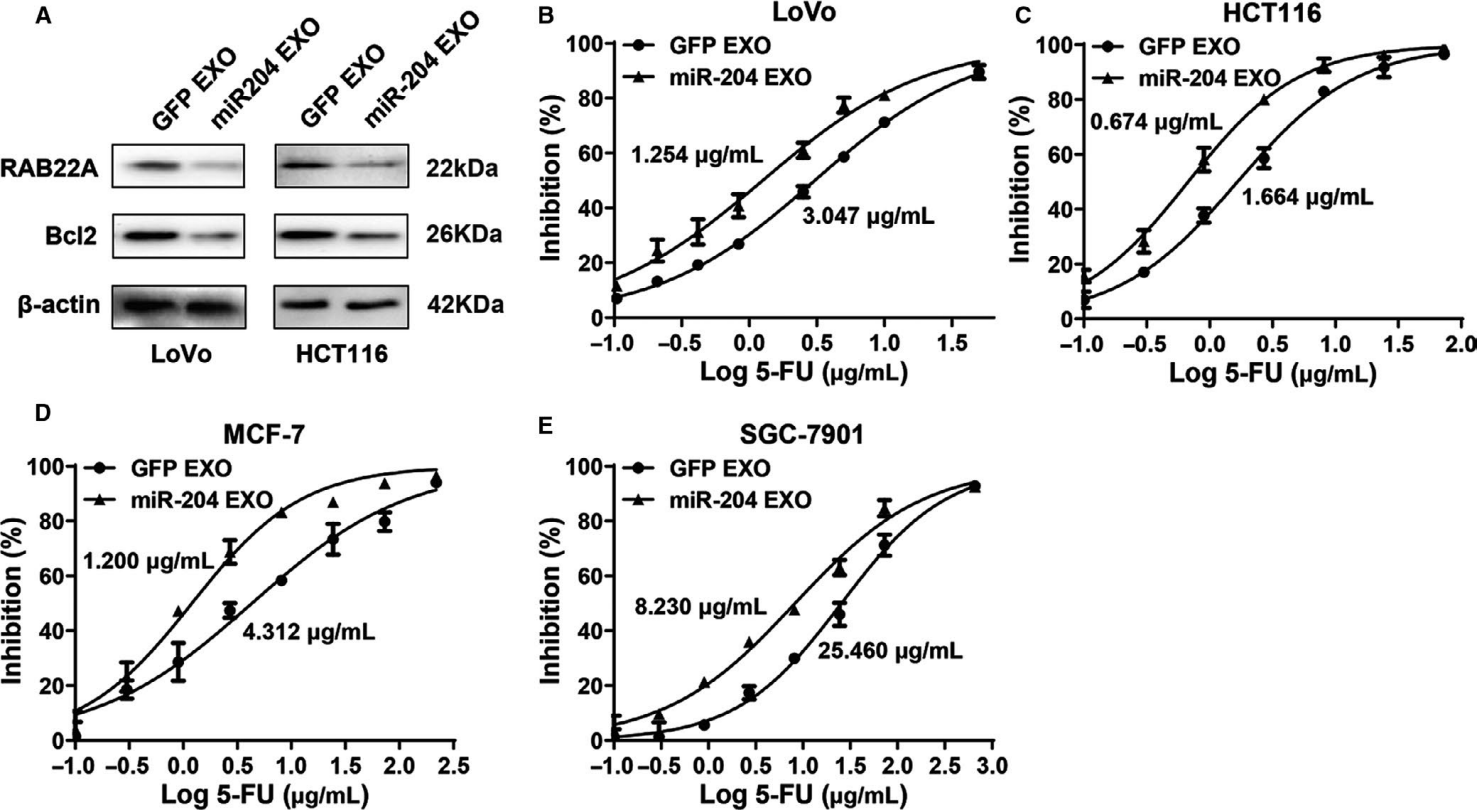
Exosomal miR‐204‐5p increases sensitivity of cancer cells to chemotherapy. A, The protein expression of miR‐204‐5p target genes (RAB22A and Bcl2) in CRC cells incubated with the exosomes of 293T‐GFP (GFP EXO) and 293T‐miR‐204 cells (miR‐204 EXO) for 48 hours were tested by Western blot. B‐E, Exosomal miR‐204‐5p increased the sensitivity of cancer cells to 5‐FU. The IC50s of LoVo (1.254 *vs* 3.047 µg/mL), HCT116 (0.674 *vs* 1.664 µg/mL), MCF‐7(1.200 *vs* 4.312 µg/mL) and SGC‐7901 (8.230 *vs* 25.460 µg/mL) cocultured with exosomal miR‐204‐5p were significantly lower than those of their corresponding controls (*P* < .01). Shown are mean ± SEM from three independent experiments

We previously found that miR‐204‐5p could suppress CRC cell proliferation and chemoresistance by targeting Bcl2 and RAB22A.^8^, ^9^ The expression of Bcl2 and RAB22A was obviously decreased in CRC cells cocultured with the 293T‐miR‐204‐derived CM or exosomes (Figure 4A). Together, these data suggested that miR‐204 EXO exerts its anticancer effects by specifically inhibiting the target genes of miR‐204‐5p.

### 3.5 Exosomal miR‐204‐5p increases the chemotherapy sensitivity of cancer cells

We previously revealed that miR‐204‐5p could inhibit chemoresistance in CRC cells.^8^, ^9^ Here, we tested whether miR‐204 EXO had a chemosensitization effect using a commonly used chemotherapeutic drug—5‐FU as a model drug. After treatment with GFP EXO or miR‐204 EXO for 48 hours, the IC50 of miR‐204 EXO‐treated CRC cells was clearly smaller than that of the control cells (Figure 4B and C). For 5‐FU is also commonly used for the treatment of gastric and breast cancers, we further checked the effects of miR‐204 EXO on the sensitivity of SGC‐7901 and MCF‐7 cells to 5‐FU. The results confirmed the chemosensitization effect of exosomal miR‐204‐5p in these cancer cells (Figure 4D and E). These data suggested that exosomal miR‐204‐5p strongly increases the chemosensitivity of cancer cells.

### 3.6 Exosomal miR‐204‐5p inhibits the tumor growth in mice

**Figure 5:**
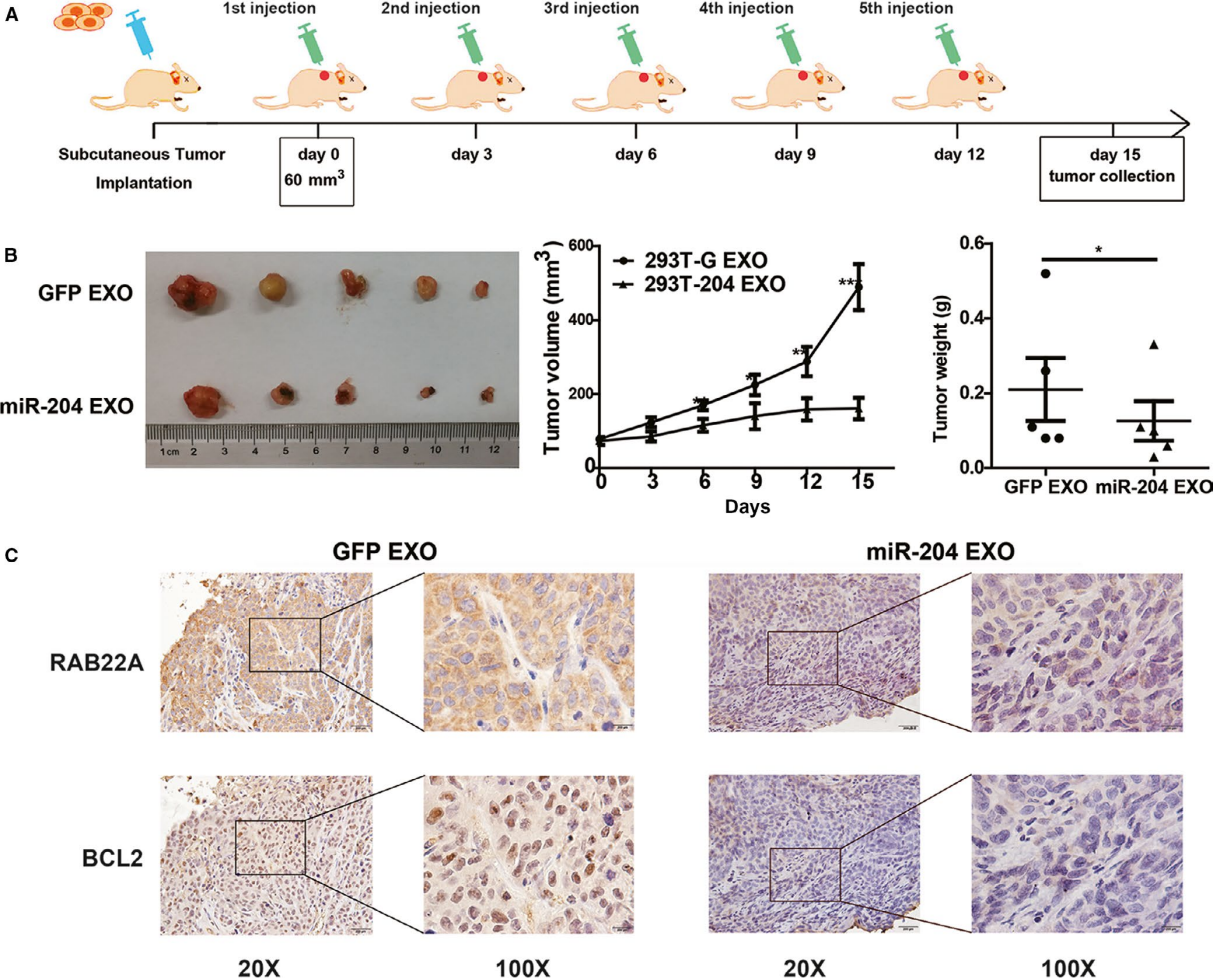
Exosomal miR‐204‐5p inhibits CRC growth in vivo. A, The schematic outline of the experimental design for the exosomal miR‐204‐5p treatments in mouse. HCT116 (2 × 10^6^) cells were injected subcutaneously into the right flank of each nude mouse. A week later, the volumes of tumors were about 60 mm^3^. GFP EXO or miR‐204 EXO was respectively injected into the xenograft tumors once every three days. These mice were sacrificed 3 days after the fifth injection. The volume and weight of mice treated with exosomal miR‐204‐5p were reduced compared with the control group. B, The effect of exosomal miR‐204‐5p therapy on the tumor growth in a nude mouse xenograft model. C, RAB22A and Bcl2 protein levels were downregulated in xenograft tissues treated with miR‐204 EXO compared with the control. *P < .05, **P < .01, ***P < .001

To further evaluate the in vivo therapeutic potential of exosomal miR‐204‐5p, we constructed a xenograft mouse model using HCT116 cells (Figure 5A). Consistent with the in vitro results, miR‐204 EXO significantly suppressed the growth rate of xenograft tumors compared with GFP EXO (Figure 5B). In addition, IHC staining showed that the protein expression of two representative targets of miR‐204‐5p (RAB22A and Bcl2) was significantly downregulated in the xenograft tissues treated with exosomal miR‐204‐5p (Figure 5C).

## 4 DISCUSSION

Exosomes can regulate the phenotypes and functions of the target cells by reprograming their signaling pathways. As important mediators of extracellular signaling, exosomes show promising clinical value for cancer diagnosis and therapy.^20^ Because of their powerful ability to deliver functional molecules into cells, stability in blood, innate biocompatibility and immune‐tolerances, exosome‐based drug delivery systems have many advantages over conventional drug delivery systems. In this study, we constructed a 293T cell line stably secreting exosomal miR‐204‐5p, and showed that exosomal miR‐204‐5p could inhibit tumor growth and reverse chemoresistance efficiently.

A key concerning systemic therapy is the nonspecific distribution of anticancer drugs. Targeted delivery of anticancer drugs can significantly increase the therapeutic effects of these drugs and at the same time decrease their toxic side effects by avoiding their accumulation in healthy organs and tissues. Many nanoparticles have been designed for drug delivery, and nanoscale therapeutics have shown promising prospects in cancer treatment.^17^ As a cell‐derived nanoparticle, exosome has attracted wide attention for its advantages over synthetic nanoparticles, and targeted exosomal delivery systems for precision nanomedicine has increasingly been regarded as new hot spots of cancer targeted‐therapy.^3^ For example, Yang et al revealed that exosome‐encapsulated anticancer drugs could cross the blood‐brain barrier and inhibit brain cancer growth.^8^ In addition to encapsulating cytotoxic agent, exosome is also a highly promising delivery vector for gene therapy.^9^, ^20^, ^21^

MiRNAs have extensive regulatory functions, and aberrant miRNA expression is closely with the development and progression of malignant neoplasms. Therefore, miRNA replacement therapies appear to be promising strategies for cancer treatment. The first miRNA‐based clinical trial was withdrawn due to toxic side effects.^22^ Suitable delivery vector is the key to successful miRNA‐based therapies. For their innate cell‐binding capacity and excellent stability, exosome is an excellent miRNA delivery system. Ohno et al obtained exosomes derived from HEK‐293 cells stable expressing the GE11 peptide or EGF, then exogenous let‐7a was transfected into these modified exosomes. Using EGFR‐expressing breast cancer xenograft model, they showed that GE11‐positive exosomes containing let‐7 suppress tumorigenesis.^5^ In this study, we constructed a 293T cell model that stably expresses high levels of miR‐204‐5p. Importantly, we showed that miR‐204‐5p could be efficiently packaged into exosomes and was delivered into cancer cells. Compared to the in vitro transfection loading method, our system could produce miR‐204‐5p‐enriched exosomes directly instead of the complicated in vitro RNA transfection procedure, avoiding of the potential toxicity and efficiency concerns of transfection.

As a powerful tumor suppressor, miR‐204‐5p is a potential therapeutic molecule for cancers. Our in vitro and in vivo assays demonstrated that the miR‐204‐5p‐enriched exosomes derived from 293T cells could inhibit cancer growth in varied types of human cancers. In a recent study, Zheng et al constructed miR‐204‐5p‐loaded PLGA/PLA‐PEG‐FA nanoparticles (FA‐NPs‐miR‐204) using surface‐functionalizing technique, and showed that FA‐NPs‐miR‐204 could be efficiently taken up by CRC cells. Functional assays demonstrated that the nanoparticle delivered miR‐204‐5p could inhibit CRC cell proliferation and promote apoptosis.^23^ However, miR‐204‐5p needs to be loaded into the nanoparticles using RNA transfection procedures.

Multidrug resistance (MDR) is the key reason responsible for cancer chemotherapy failure. Our and other studies have demonstrated that miR‐204‐5p could increase the sensitivity of tumor cells to chemotherapeutic drugs.^8^, ^9^, ^23^, ^24^ In this study, our in vitro assays confirmed that exosomal miR‐204‐5p could efficiently enhance the cytotoxicity of 5‐FU by increasing 5‐FU‐induced apoptosis. Liu et al showed that engineered tumor‐targeting hEVs are promising natural vectors to overcome MDR.^25^ In addition, a recent study reported that exosomal miR‐128‐3p could increase the chemosensitivity of CRC cells to oxaliplatin.^26^ These data suggested that combined with conventional chemotherapies, exosome delivered miR‐204‐5p may overcome MDR and improve prognosis of cancer patients.

Taken together, in this study, we developed a new miRNA delivery vehicle based on the 293T cells stably secreting miR‐204‐5p‐enriched exosomes. Functional assays confirmed that exosomal miR‐204‐5p could efficiently inhibit cancer cell proliferation, induce apoptosis, and increase chemosensitivity in cancer cells. There are several limitations that should not be neglected before considering the clinical translation of our exosomal miR‐204‐5p therapeutic system. First, more attention should be paid to increase the specificity of exosomes to cancer cells in the future work probably by suitable targeting modifications of exosomes. Second, the safety of the exosome delivery system should be evaluated. Finally, the manufacturing, storage, and administration of these therapeutic exosomes should be evaluated in detail in the future.

## 5 CONCLUSIONS

In this study, we developed a new miRNA delivery vesicle based on the HEK293T cells stably secreting miR‐204‐5p‐enriched exosome. Functional assays confirmed that exosomal miR‐204‐5p could efficiently inhibit cancer cell proliferation, promote apoptosis and increase chemosensitivity by specific suppressing the targets of miR‐204‐5p. This approach is potentially applicable to other gene drug delivery methods mediated by exosomes.

## CONFLICT OF INTEREST

All authors declare that they have no conflict of interest.

## AUTHOR CONTRIBUTIONS

Yao SR contributed to the data acquisition and analysis; Huang ZH, Yin Y, and Wu ZM contributed to the study conception and design; Jin GY, Li D, Li M, Hu Y, Feng YY, Bian ZH, Zhang J, and Mao Y helped collect the data; Huang ZH, Yao SR and Yin Y wrote the manuscript; all authors read and approved the final manuscript.

## Data Availability

The data supporting the research results can be obtained from the corresponding authors based on reasonable requirements.
